# Supplementary material for: Comprehensive Annotation and Functional Exploration of MicroRNAs in Lettuce
Source: Front Plant Sci. 2021 Dec 24;12:781836. doi: 10.3389/fpls.2021.781836 (PMC8739914; doi:10.3389/fpls.2021.781836)
Supplement: Supplementary file 2 [file Data_Sheet_8.ZIP › GSM2230750.plot/Lsa-miR168b_Lsat_1_v5_gn_2_19760.1_1492_TPlot.pdf]

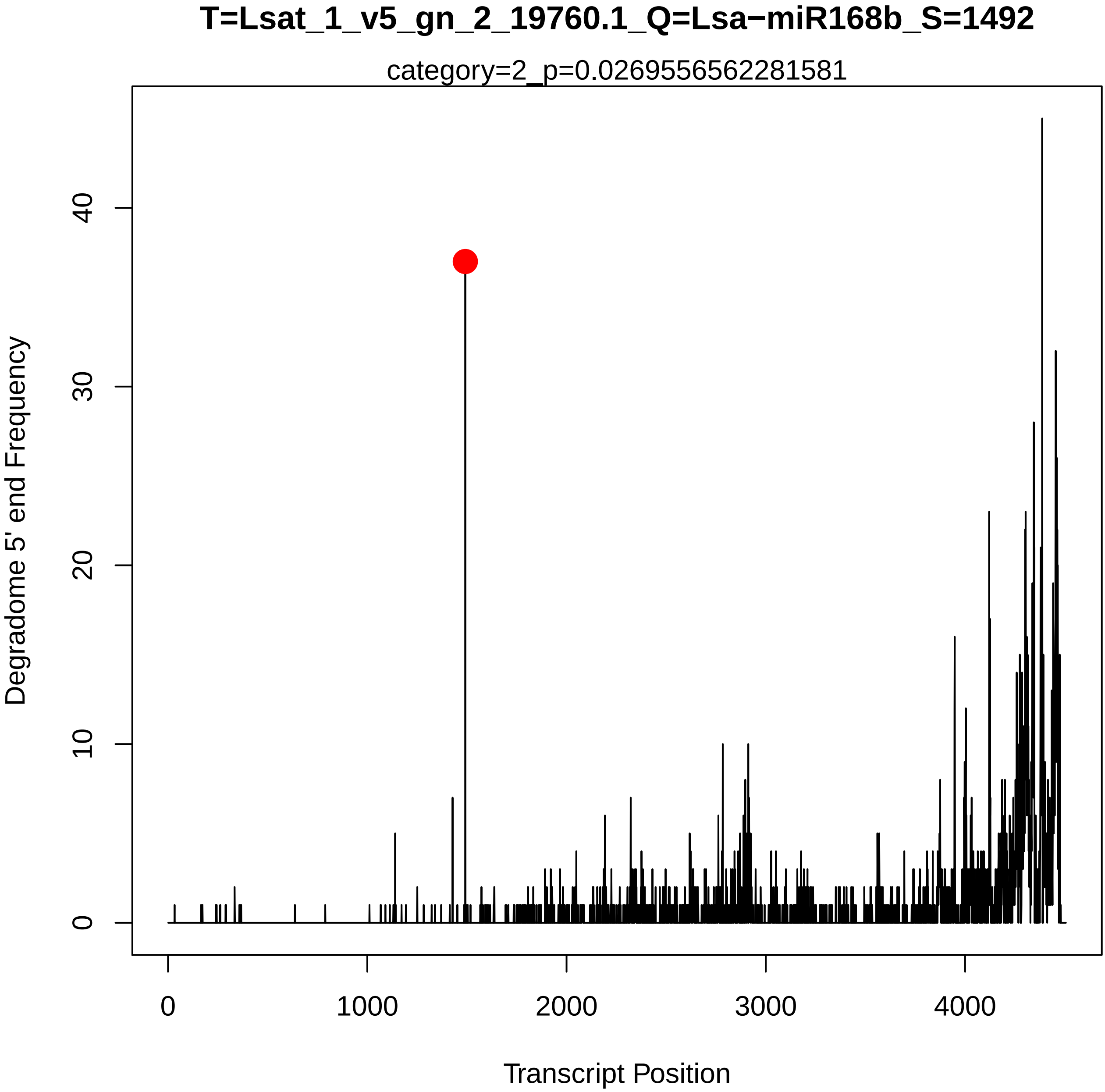
T=Lsat_1_v5_gn_2_19760.1_Q=Lsa−miR168b_S=1492
category=2_p=0.0269556562281581
Degradome 5' end Frequency
0
10
20
30
40
0
1000
2000
3000
4000
Transcript Position
